# Interleukin-4 Boosts Insulin-Induced Energy Deposits by Enhancing Glucose Uptake and Lipogenesis in Hepatocytes

**DOI:** 10.1155/2018/6923187

**Published:** 2018-11-21

**Authors:** Ching-Ping Yang, Ming-Yuh Shiau, Yi-Ren Lai, Kuo-Ting Ho, Chiao-Wan Hsiao, Chun-Jung Chen, Yu-Li Lo, Yih-Hsin Chang

**Affiliations:** ^1^Department of Medical Research, Taichung Veterans General Hospital, Taichung, Taiwan; ^2^Department of Nursing, College of Nursing, Hungkuang University, Taichung, Taiwan; ^3^Department of Biotechnology and Laboratory Science in Medicine, National Yang-Ming University, Taipei, Taiwan; ^4^Program in Molecular Medicine, National Yang-Ming University and Academia Sinica, Taiwan; ^5^Department and Institute of Pharmacology, National Yang-Ming University, Taipei, Taiwan

## Abstract

Type 2 diabetes mellitus (T2DM), with dysregulated hepatic gluconeogenesis as the major cause of fasting hyperglycemia, is closely associated with chronic inflammation. We previously demonstrated interleukin-4 (IL-4) improves insulin sensitivity and glucose tolerance while reducing lipid deposits. The present study examined the *in vitro* effects of IL-4 on insulin signaling molecules, glucose uptake, and lipid metabolism in hepatocytes, as well as *in vivo* effects on hepatic adiposity, for elucidating the roles of IL-4 in hepatic energy metabolism. Potential interaction between IL-4 and insulin in regulating hepatic metabolism was also investigated. Our results showed that IL-4 enhanced Akt and GSK-3*α*/*β* phosphorylations, which in turn promoted glycogen synthesis. IL-4 not only potentiated basal glucose uptake by upregulating glucose transporter 2 expression but also promoted insulin-induced glucose uptake. Additionally, IL-4 increased triglyceride contents through facilitating free fatty acid uptake and expression/activity of lipogenic enzymes. The major effects of IL-4 on the liver were to promote energy storage by boosting insulin-stimulated glucose uptake and lipid synthesis. This study provides evidence to implicate the novel roles of IL-4 in mediating hepatic glucose and lipid metabolism, interactions between immune responses and metabolic homeostasis, and the involvement of IL-4 in metabolic abnormalities.

## 1. Introduction

Type 2 diabetes mellitus (T2DM) is a common endocrine disease. The etiology leading to this metabolic disease is still an enigma although insulin resistance has been implicated to play an important role [1]. The concept of interaction between inflammation and metabolic abnormalities is initiated by Hotamisligil et al. [2]. They demonstrate that proinflammatory cytokine tumor necrosis factor-*α* (TNF-*α*) is markedly increased in adipocytes of obese animals. From then on, accumulating studies prove that T2DM is an inflammatory condition characterized by elevated acute phase inflammatory reactants in the plasma [3–6]. Accordingly, excess glucose and macronutrient intake can produce oxidative stress which then results in the increased circulatory proinflammatory cytokines, such as TNF-*α* and interleukin-6 (IL-6). These upregulated cytokines, together with the excess free fatty acids (FFAs) released from adipose tissue into the bloodstream and liver, impair insulin sensitivity and induce hepatic gluconeogenesis.

The liver plays a critical role in maintaining glucose homeostasis through the finely tuned regulation of gluconeogenesis and glycogen synthesis. Dysregulated hepatic glucose output is the major cause of fasting hyperglycemia in diabetic patients [7, 8]. The increased postprandial insulin not only enhances glucose uptake ability in muscle and adipose tissues but also inhibits the expression of hepatic genes responsible for gluconeogenesis, such as phosphoenolpyruvate carboxykinase (PEPCK) [9–11]. On the contrary, the postprandial expression of the gluconeogenic enzymes is increased in T2DM patients due to insulin resistance [12]. Expression of glycogenic serine/threonine protein kinase glycogen synthase kinase 3 (GSK-3) is also upregulated in hepatocytes among individuals with insulin resistance and T2DM [13–15]. The dysregulated gluconeogenic and glycogenic enzymes further exacerbate the metabolic abnormalities in diabetic patients.

As mentioned above, T2DM is closely related to chronic inflammation. Interleukin-1 (IL-1), IL-6, and TNF-*α* are proved to impair insulin action on peripheral glucose consumption and hepatic glucose output [16–19], which suggests that cytokines are involved in the decreased insulin sensitivity. While much is known about the effects of Th2-derived IL-6 on glucose metabolism, it is of interest to explore possible participation and regulation of other Th2 cytokines in metabolic homeostasis. We previously reported that *IL-4* genotypes are significantly associated with T2DM and high-density lipoprotein-cholesterol (HDL-C) [20]. Significant association between genetic polymorphisms of the IL-4 receptor *α* chain (IL-4R*α*) and HDL is also identified [21]. Moreover, IL-4 improves insulin sensitivity and glucose tolerance while inhibiting lipid accumulation which leads to decreased fat mass [22, 23]. Our most recent results show that IL-4 harbors prolipolytic capacity by inhibiting adipogenesis and lipid accumulation as well as promoting lipolysis in mature adipocytes to reduce lipid deposits [24, 25]. The above results not only uncover novel roles of IL-4 in regulating glucose/lipid metabolism but also reveal the involvement of IL-4 in metabolic abnormalities such as obesity and T2DM.

To further address the roles of IL-4 in energy metabolism and pathogenesis of obesity and T2DM, the present study examined the effects of IL-4 on glucose and lipid metabolism in hepatocytes. Our data show that IL-4 not only potentiates insulin-independent basal glucose uptake by upregulating hepatic glucose transporter 2 (GLUT2) expression but also promotes insulin-induced glucose uptake and glycogen synthesis. Additionally, IL-4 synergizes insulin-stimulated hepatic FFA uptake for *de novo* lipogenesis. The net effect of IL-4 on energy metabolism is to aid insulin-dependent energy deposits in hepatocytes.

## 2. Materials and Methods

### 2.1. Materials

Reagents were obtained from the following sources: antibodies against Akt, phospho-Ser473 Akt, GSK-3*α*/*β*, and phosphor-GSK-3 *α*/*β* Ser21/9 from Cell Signaling Technology (Danvers, MA, USA); anti-ACC1 and anti-DGAT2 from Gentex Inc. (Irvine, CA, USA); anti-IL-4R from Abcam (San Francisco, CA, USA); mouse IL-4 from Millipore (Temecula, CA, USA); ECL reagent from Calbiochem (Merck Millipore, Billerica, MA, USA); insulin, anti-GATA3, anti-*β*-actin, fatty acid uptake, and glycogen assay kits from Sigma (St. Louis, MO, USA); anti-phospho-STAT-6 from Millipore Corporation; anti-SREBP-1, anti-PPAR*α*, anti-GAPDH, anti-GLUT2, and anti-PEPCK from Santa Cruz Biotechnology Inc.; TRIzol Reagent from Life Technologies (Carlsbad, CA, USA); anti-FAS from BD Biosciences; triglyceride quantification kit from BioVision Inc. (Milpitas, CA, USA).

### 2.2. Cell Culture and Treatments

Human HepG2 and Huh7 hepatocytes were cultured in DMEM (GIBCO) containing 1% penicillin, streptomycin, and 10% fetal bovine serum at 37°C in a 5% CO_2_ atmosphere. For IL-4 and/or insulin treatment, after 4 hours of serum starvation, cells were treated with 10 ng/ml IL-4 [22–24] and/or 100 nM insulin for the time indicated.

### 2.3. RNA Extraction and RT-PCR

Total cellular mRNA was extracted using TRIzol Reagents. Briefly, cDNA was synthesized using total mRNA, oligo dT primer, and 5x MMLV RT. One *μ*g of synthesized cDNA was then amplified using target sequence-specific primer sets (IL-4R: 5′-GGAAGAGGGGTATAAGCCTTT-3′ and 5′-CACGGAGACAAAGTTCACGAT-3′ and GAPDH: 5′-ACCACAGTCCATGCCATCAC-3′ and 5′-TCCACCACCCTGTTGCTGTA-3′). All PCR reactions were carried out by initial denaturation for 5 min at 95°C, followed by 30 cycles consisting of 95°C for 1 min, annealing for 1 min, and 72°C for 1 min. The PCR products were electrophoresed and visualized by ultraviolet transilluminator.

### 2.4. Western Blot

Total cell lysates were extracted at 4°C by a lysis buffer containing proteinase and phosphatase inhibitors. Extracts were centrifuged at 14,000 rpm at 4°C for 15 min, and supernatants were collected. Protein extract samples (40 *μ*g) were resolved by SDS-PAGE and electrotransferred to a PVDF membrane. Membranes were permeated with a TBST buffer, incubated with primary antibodies, followed by HRP-conjugated secondary antibodies (ZYMED Laboratories Inc. & NEN, Boston, USA). Proteins were visualized using ECL reagents and quantitated by densitometry.

### 2.5. Glucose Uptake Fluorometric Assay

The glucose uptake assay was performed after cells were incubated with a glucose-free KRPH buffer for 3 h. Cells were treated with IL-4 and/or insulin for 20 min and fed with 100 *μ*mol/l 2-[N-(7-nitrobenz-2-oxa-1,3-diazol-4-yl)amino]-2-deoxy-D-glucose (2-NBDG). Cellular 2-NBDG uptake was terminated after 10 min by an ice-cold KRPH buffer containing 10 mM glucose. Cells were washed and lysed, and intracellular fluorescence intensity was measured (485/540 nm, Infinite 200).

### 2.6. Glycogen Synthesis Assay

Cells were treated with IL-4 and/or insulin for 16 hrs after serum starvation, homogenized, and boiled for 5 min. Supernatants were collected after the homogenates were centrifuged at 13,000g for 5 min. Each of 10x diluted samples by Hydrolysis Buffer in 96-well microplate was incubated with Hydrolysis Enzyme Mix for 30 min, followed by incubation with Reaction Mix for another 30 min at room temperature. Glycogen contents were measured by the absorbance at 570 nm.

### 2.7. Lipid Accumulation, Fatty Acid Synthesis and Uptake, and Triglyceride Assay

Oil Red O staining was performed to analyze hepatic lipid accumulation [24]. For fatty acid synthesis, endogenous FFA synthesis was induced by adding acyl-CoA synthesis reagents in cell lysates after the cells were exposed to IL-4 and/or insulin treatment for 24 hours after serum starvation. The fatty acid contents were then measured. For fatty acid uptake, cells were incubated with 100 *μ*l fluorescent TF2-C12 fatty acid-containing dye-loading solution for 60 min after IL-4 and/or insulin treatment. The intracellular fatty acids were then quantitated by measuring fluorescence intensity. For the triglyceride assay, cells were homogenized and heated to dissolve cellular triglycerides. Supernatants were collected after the homogenates were centrifuged at 13,000g for 2 min. Then each of 10x diluted samples by Triglyceride Assay Buffer in 96-well microplate was incubated with 2 *μ*l lipase for 30 min, followed by incubation with Reaction Mix for another 30 min at room temperature. Triglyceride contents were measured by the absorbance at 570 nm.

### 2.8. Animal Experiments

Animal experiments were conducted as described. In brief, 8-week-old male C57BL/6 mice were i.p. injected twice with AdIL-4 or AdLacZ, followed by i.p. streptozotocin (STZ; 100 mg/kg; Sigma-Aldrich, St Louis, MO, USA) administration to induce the type 2 diabetic onset [26]. For high-fat diet (HFD) experiments, 4-week-old male C57BL/6 mice were fed with HFD or standard chow diet and i.p. administered with recombinant IL-4 (1000 pg per mouse) every other day for 8 weeks as described [22]. Animal protocols were reviewed and approved by the Institutional Animal Care and Use Committee, National Yang-Ming University.

### 2.9. Statistical Analysis

Results were presented as mean ± SEM, and the significant difference between groups was analyzed by one-way or two-way analysis of variance using SPSS software. Statistical difference was defined as *p* < 0.05 for all test.

## 3. Results

### 3.1. IL-4 Signaling Is Successfully Transduced into Hepatocytes

**Figure 1:**
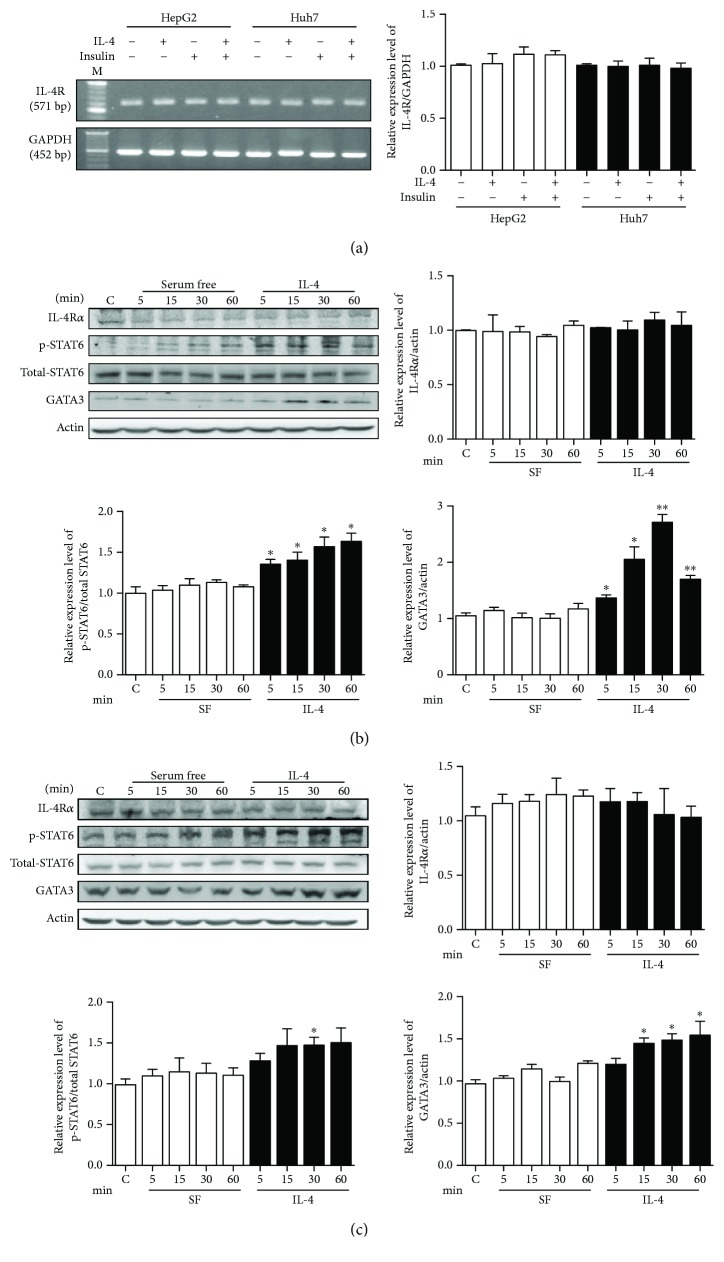
IL-4 signaling is successfully transduced in hepatocytes. (a) IL-4R*α* mRNA was analyzed in HepG2 and Huh7 cells by RT-PCR. Cells were serum starved, followed by IL-4 and/or insulin treatment for 24 hrs. Quantitative results were shown in the right panel. (b and c) IL-4R*α*, p-STAT6, and GATA3 were analyzed by Western blotting in HepG2 (b) and Huh7 (c). Cells were serum starved (SF), followed by IL-4 and/or insulin treatment for the indicated time. Bar graphs showed the corresponding quantitative results (*n* = 3). ^∗^ *p* < 0.05 vs. control; ^∗∗^ *p* < 0.01 vs. control.

Expressions of the IL-4 receptor (IL-4R*α*), downstream signaling molecule signal transducer and activator of transcription 6 (STAT6), and targeting gene GATA-binding protein 3 (GATA3) were first determined in HepG2 and Huh7 hepatocytes to confirm the transduction of IL-4 signals. As shown in Figure 1(a), IL-4R*α* mRNA was stably expressed in both cells. Phosphorylated STAT6 (p-STAT6) and GATA3 were increased in HepG2 (Figure 1(b)) and Huh7 (Figure 1(c)) cells under IL-4 treatment. These findings indicate that IL-4 signaling can be successfully triggered and transduced in hepatocytes.

Intriguingly, GATA3 at 60 min of IL-4 treatment was decreased compared to 30 min of IL-4 treatment in HepG2 (Figure 1(b)) while its expression maintained increased at 60 min in Huh7 (Figure 1(c)). We speculated that downregulation and/or negative feedback of the signaling machinery in HepG2 cells and/or the intrinsic delicate differences between these two cell lines may explain the differential GATA3 expression pattern in the presence of the IL-4 stimulus.

### 3.2. IL-4 Performs Synergistic Activity to Boost Insulin Signaling

Insulin promotes glucose uptake in its targeting cells to reduce blood glucose [27]. In addition to the central insulin signaling molecule AKT, GSK-3 plays an important regulatory role in glycogen synthesis [28, 29]. Postprandial insulin signaling inhibits GSK-3 phosphorylation, which in turn results in glycogen synthase (GS) activation and glycogen synthesis. Total and active forms of GSK-3 are associated with insulin resistance and highly expressed in T2DM [30]. In this context, the putative regulation of AKT and GSK-3*α*/*β* activity by IL-4 was examined to explore the possible effects of IL-4 on glucose metabolism and glycogen synthesis. In addition, putative interaction between insulin and IL-4 in regulating hepatic energy metabolism was also examined.

**Figure 2:**
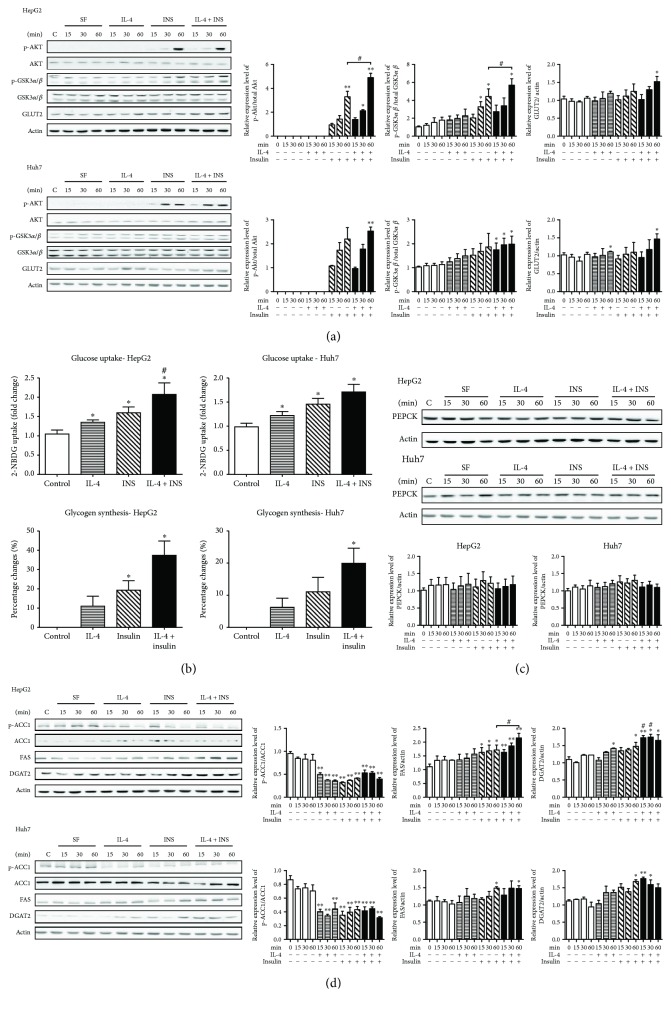
IL-4 performs synergistic activity to boost insulin signaling, promoting insulin-triggered hepatic glucose uptake, glycogen synthesis, and fatty acid synthesis. HepG2 and Huh7 cells were serum starved, followed by IL-4, insulin (INS), or combined treatment (IL-4 + INS) for the indicated time. Cell lysates were harvested, with p-AKT, p-GSK-3*α*/*β*, and GLUT2 analyzed by Western blotting (a). Hepatic glucose uptake and glycogen synthesis (b). PEPCK (c) p-ACC1, FAS, and DGAT2 (d) analyzed by Western blotting. Bar graphs showed the corresponding quantitative results (*n* = 3). ^∗^ *p* < 0.05 vs. 0 min; ^∗∗^ *p* < 0.01 vs. 0 min; ^#^ *p* < 0.05 vs. INS at the same time point.

Phosphorylated AKT (p-AKT) and GSK-3*α*/*β* (p-GSK-3*α*/*β*) were analyzed in cells under IL-4, insulin (INS), or combined (IL-4 + INS) treatment after serum starvation (SF). As expected, insulin markedly promotes p-AKT and p-GSK-3*α*/*β* in HepG2 and Huh7 (Figure 2(a)) cells. While p-AKT and p-GSK-3*α*/*β* were not apparently affected by IL-4, IL-4 boosted insulin-induced p-AKT and p-GSK-3*α*/*β*. GLUT2 is the major hepatic glucose-sensing and transporting protein [31, 32]. Interestingly, IL-4 upregulated GLUT2 expression while insulin did not cause prominent alterations. The above results reveal that IL-4 plays a synergistic role in insulin signaling through AKT and GSK-3*α*/*β* in hepatocytes. It suggests that the IL-4-improved glucose tolerance results at least in part from enhancing insulin action via AKT in hepatocytes. Intriguingly, IL-4 exhibits insulin-independent activity to enhance hepatic GLUT2 expression.

### 3.3. IL-4 Promotes Hepatic Glucose Uptake and Glycogen Synthesis

According to the above findings, we hypothesized that IL-4 mediated glucose metabolism and glycogen synthesis via the insulin signaling pathway. Therefore, glucose uptake and glycogen synthesis under IL-4 exposure were subsequently examined. Intriguingly, IL-4 not only significantly enhanced intracellular 2-NBDG levels (about 1.25 folds) in the absence of insulin but also significantly enhanced insulin-induced glucose uptake (about 1.7–2 folds) (Figure 2(b)). It implicates that IL-4 harbors blood glucose-lowering activity by promoting both basal and insulin-induced glucose uptakes. We suggest that IL-4 may promote basal hepatic glucose uptake via upregulating GLUT2 expression. These results also support our previous *in vivo* findings that IL-4 improves insulin sensitivity and glucose tolerance by upregulating AKT phosphorylation and attenuating GSK-3 activities [22].

Glycogen synthesis in hepatocytes can be divided into 2 major steps. First, insulin-induced p-AKT inhibits GSK-3*α*/*β* activity, which then upregulates GS to trigger glycogen synthesis. Second, the GLUT2-mediated uptake of glucose is phosphorylated to glucose-6-phosphate, which serves as the substrate for glycogen synthesis. The data that IL-4 regulated GSK-3*α*/*β* activity (Figure 2(a)) and glucose uptake (Figure 2(b)) implied IL-4 also modulated glycogen synthesis. Therefore, glycogen contents after IL-4 and/or insulin treatment were investigated. While intracellular glycogen contents were slightly increased by IL-4, they were markedly elevated by combined treatment (Figure 2(b)). It suggests that IL-4 also boosts insulin-stimulated glycogen synthesis through regulating GSK-3*α*/*β* activity and results in increased hepatic energy deposits.

Disturbance of hepatic gluconeogenesis results in the deterioration of diabetic hyperglycemia. We next examined if IL-4 also modulates the rate-limiting enzyme of gluconeogenesis, PEPCK. The results showed that PEPCK remained on a constant level under IL-4 and/or insulin exposure (Figure 2(c)) in HepG2 and Huh7 cells. Combining the above results, IL-4 exerts positive regulatory capacity to glucose metabolic machinery and promotes insulin efficacy in hepatocytes. In addition, IL-4 may deviate hepatic metabolism towards energy deposits since IL-4 upregulates GLUT2 and basal glucose uptake in the absence of insulin.

### 3.4. IL-4 Promotes Insulin-Triggered Hepatic Lipid Deposits

Our previous study demonstrates that IL-4 participates in lipid metabolism by inhibiting lipid deposits of adipose tissues, which lead to decreased weight gain and fat mass [22]. Besides, IL-4 harbors pro-lipolysis capacity by inhibiting adipocyte differentiation and lipid accumulation in mature adipocytes [24, 25]. In this context, the possible regulation of IL-4 to hepatic lipid metabolism was next investigated.

**Figure 3:**
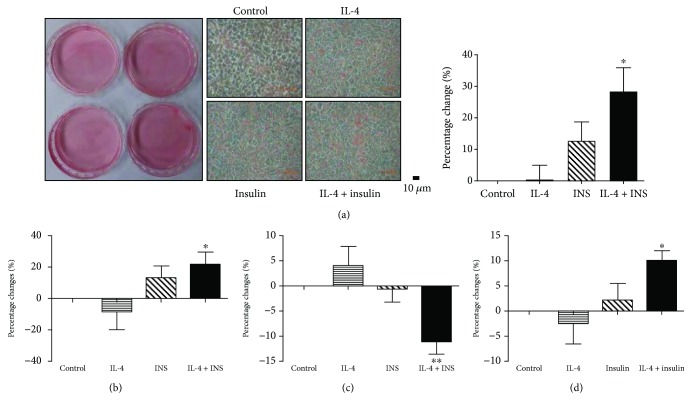
IL-4 promotes insulin-triggered hepatic lipid deposits. (a and b) HepG2 cells were serum starved, followed by IL-4 and/or insulin treatment for 72 hrs. Intracellular lipid and triglyceride contents were analyzed by Oil Red O staining (a) and the triglyceride assay kit (b). (c) HepG2 cells were serum starved, followed by inducing FFA synthesis under IL-4 and/or insulin for 24 hrs. Then FFA levels were measured. (d) HepG2 cells were serum starved, followed by IL-4 and/or insulin treatment for 60 min. Then cells were incubated with fluorescent fatty acid dye-loading solution for 60 min; then fluorescence intensity was measured. Bar graph in (a) showed the corresponding quantification of the staining results, and (b–d) showed the percentage of changes (*n* = 3). ^∗^ *p* < 0.05 vs. control; ^∗∗^ *p* < 0.01 vs. control.

Putative alterations of the important enzymes for triglyceride synthesis, including acetyl-CoA carboxylase 1 (ACC1), fatty acid synthase (FAS), and diglyceride acyltransferase 2 (DGAT2), under IL-4 and/or insulin treatment were analyzed. As shown in Figure 2(d), IL-4 significantly downregulated p-ACC1 but showed no effects on FAS and DGAT2, except for DGAT2 after 60 min in HepG2. IL-4 also boosted insulin-stimulated FAS and DGAT2 in HepG2 cells. The results suggest that IL-4 exhibits synergistic activity to enhance the insulin-induced triglyceride synthesis through regulating ACC1, FAS, and DGAT2. Therefore, the hepatic lipid contents were examined to verify the above hypothesis. While the lipid contents were not apparently altered by IL-4, they were significantly increased by about 30% under combined treatment (Figure 3(a)). Intracellular triglycerides were further measured to confirm the observations. In consistent with the Oil Red O staining data, Figure 3(b) revealed that triglyceride contents under combined treatment were significantly increased by about 30%. It indicates that IL-4 shows synergistic activity to facilitate insulin efficacy for increasing hepatic lipid storage.

Body lipid reservoir is controlled by a finely orchestrated mechanism. The major sources of circulatory FFAs are diet, endogenous synthesis, and peripheral tissues, with adipose tissue and the liver as the major organs to keep the dynamic balance. Under the status of ambient nutrition, excess nutrients are stored in adipocytes as triglycerides, and the elevated circulatory FFAs are transported into the liver [33]. The hepatic FFAs either undergo *β*-oxidation for generating energy or are packed as triglycerides then stored in the liver or released into the bloodstream as energy sources for other cells. It was intriguing for us to examine if the increased hepatic triglyceride deposit by combined treatment was originated from endogenous FFA synthesis or the uptake of exogenous FFAs.

Cells were exposed to IL-4 and/or insulin treatment for 24 hours and allowed to synthesize FFAs. Intriguingly, although FFAs were prominently decreased about 10% by combined treatment (Figure 3(c)), FFA uptake was significantly increased by about 10% (Figure 3(d)). Taking the above results together, it demonstrates that IL-4 promotes insulin-induced hepatic anabolism by upregulating FFA uptake and triglyceride synthesis. We suggest that, in response to the combined treatment, hepatocytes increase intracellular triglyceride storage through upregulated triglyceride synthesis using both the endogenous synthesized and the uptake exogenous FFAs as building blocks.

### 3.5. IL-4 Reduces Diet-Induced Obesity in Mice

**Figure 4:**
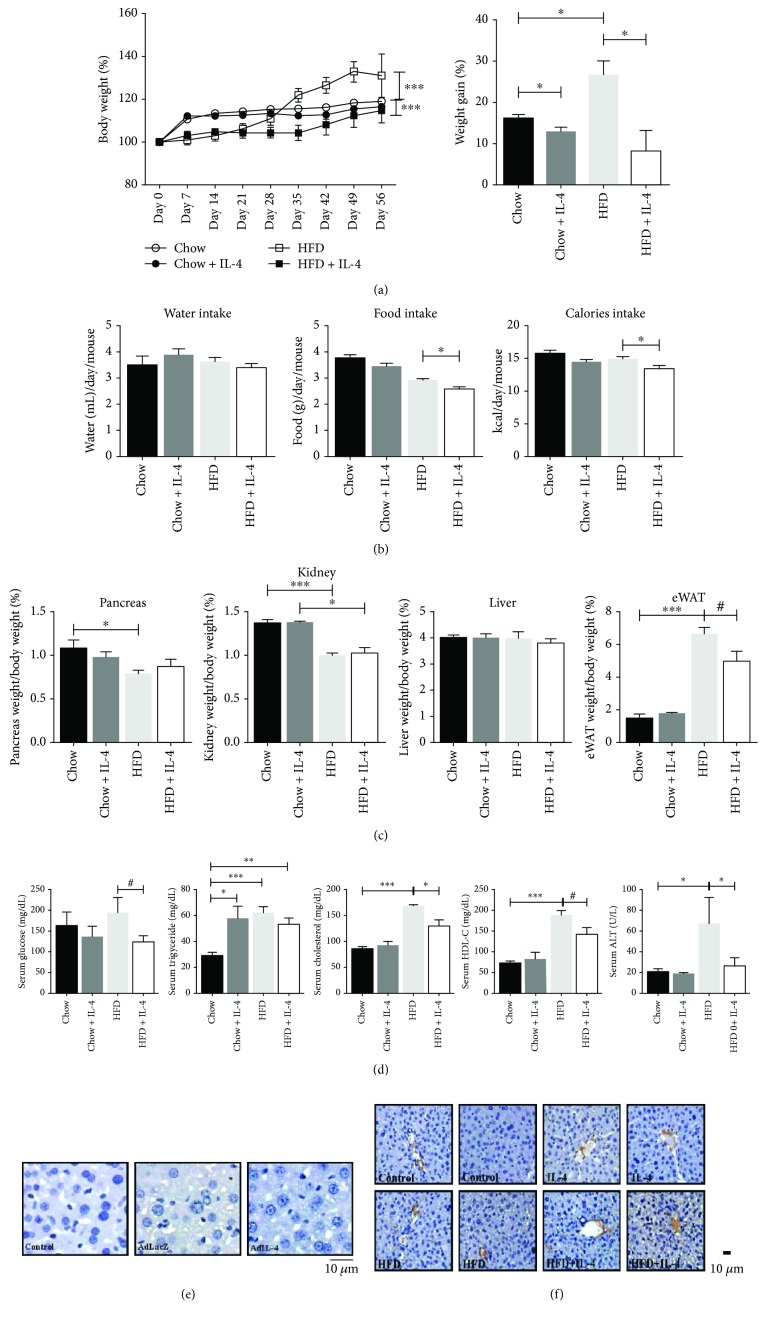
Metabolic profiles of chow diet and high-fat diet mice treated with IL-4. Results of the temporal alterations in body weight of mice receiving either IL-4 (1000 pg/mouse per two days, i.p.) or PBS fed with the HFD or chow diet: (a) body weight gain curves (left panel) and average weight gain (right panel); (b) water, foods, and caloric intakes; (c) average weight of the pancreas, kidney, liver, and eWAT. (d) Fasting serum glucose, triglyceride, cholesterol, HDL, and ALT levels were measured after 16 weeks of either HFD or chow diet with IL-4 treatment during the final 8 weeks. Data are mean ± SEM. *n* = 5–8/group. ^∗^ *p* < 0.05 vs. control; ^#^ *p* = 0.065 vs. control. The livers were obtained from STZ-induced diabetic AdIL-4 mice (e) or obesity-induced insulin resistant HFD + IL-4 mice (f). Lipid accumulation and quantification were analyzed by immunohistochemical staining of lipid droplet-specific antigen perilipin. Scale bar: 10 *μ*m. Original magnification: 400x; *n* = 5.

Results from the above *in vitro* experiments show that IL-4 regulates energy metabolism by promoting glucose uptake and lipid deposits in hepatocytes. We further examined the *in vivo* effects of IL-4 on hepatic energy metabolism. Mice were first fed with either high-fat diet (HFD) (60% kcal derived from fat) or chow diet (10% kcal derived from fat) for 16 weeks and concurrently treated with either IL-4 or PBS (chow mice) in the last 8 weeks as described [22]. Total body weight gain in HFD mice (30%) was significantly higher than the chow mice (18%) at the end of the study period (Figure 4(a)). The average weight gain in HFD mice receiving IL-4 treatment was significantly reduced to 1/3 (HFD + IL-4 mice, ~8.2%, Figure 4(a)) of that in HFD mice (~26.5%). The average weight gain in chow diet mice receiving IL-4 injection (chow + IL-4 mice) was also significantly reduced.

Additionally, the effects of IL-4 on food and water intake were investigated to explore if IL-4 modulated feeding behavior. While the water intake between chow and HFD mice was not statistically different, food intake of HFD mice with IL-4 administration was significantly reduced (Figure 4(b)). The energy intake was also significantly lowered in HFD + IL-4 mice (~13.21 kcal/mouse/day) compared to the HFD group (~14.77 kcal/mouse/day, Figure 4(b)). The capacity of IL-4 to reduce food intake through modulating the hypothalamus-released appetite-regulating hormones by downregulating the expression of orexigenic neuropeptide [agouti-related protein (AgRP) and neuropeptide-Y (NP-Y)] while increasing anorexigenic neuropeptide expression [proopiomelanocortin (POMC)] was very likely the underlying mechanism leading to the above findings (our unpublished observations). These data suggest that the reduced body weight gain in IL-4-treated mice may partly be due to the decreased food and caloric intakes (Figure 4(b)).

The weights of the liver and epididymal white adipose tissue (eWAT) were significantly increased in HFD mice compared to the chow group; whereas, no differences were observed in the weights of the liver, pancreas, and kidney (Figure 4(c)). eWAT weights in HFD + IL-4 mice were significantly decreased. Furthermore, data from blood biochemical parameters revealed that HFD mice displayed higher serum total cholesterol, triglycerides, and HDL. Intriguingly, except for the increased triglycerides in chow mice, glucose and lipid panels including cholesterol and HDL in HFD + IL-4 mice were significantly reduced (Figure 4(d)). It is well recognized that HFD triggers severe liver damage and chronic inflammation [34], leading to the elevated circulating aspartate aminotransferase (AST) and alanine aminotransferase (ALT) [35, 36]. As shown in Figure 4(d), HFD mice exhibited significantly increased serum ALT levels, while IL-4 treatment showed prominent protective effects to attenuate the HFD-induced ALT. Additionally, in support of our previous conclusion [22], both glucose tolerance test (GTT) and insulin tolerance test (ITT) showed that HFD mice with IL-4 administration exhibited better glucose tolerance and insulin sensitivity (Supplementary Figure 1). The above results indicate that IL-4 inhibits eWAT formation, regulates glucose and lipid metabolic homeostasis, and alleviates hepatic damage in HFD mice.

### 3.6. IL-4 Increases Hepatic Adiposity *In Vivo*

We further investigated the *in vivo* influences of IL-4 to the liver under diabetic and insulin resistant conditions. Results show that compared with HFD mice, IL-4 treatment exhibited improved glucose tolerance on HFD mice (data not shown). Besides, lipid contents in livers obtained from STZ-induced diabetic AdIL-4 mice (diabetes with transient IL-4 overexpression) and HFD + IL-4 mice (obesity-induced insulin resistant status with long-term IL-4 overexpression) [22] were immunohistochemically analyzed. Hepatic lipid contents remained unchanged in AdIL-4 mice (Figure 4(e)) but were increased in HFD + IL-4 mice (Figure 4(f)). Therefore, combination of HFD and long-term IL-4 administration shows a synergistic effect to increase hepatic adiposity. Besides, the data support the above *in vitro* observations that the increased hepatic adiposity may result from upregulated flux of FFAs from the periphery to the liver and enhanced hepatic lipogenesis.

### 3.7. IL-4 Promotes Gluconeogenesis and Hepatic Lipogenesis in HFD Mice

**Figure 5:**
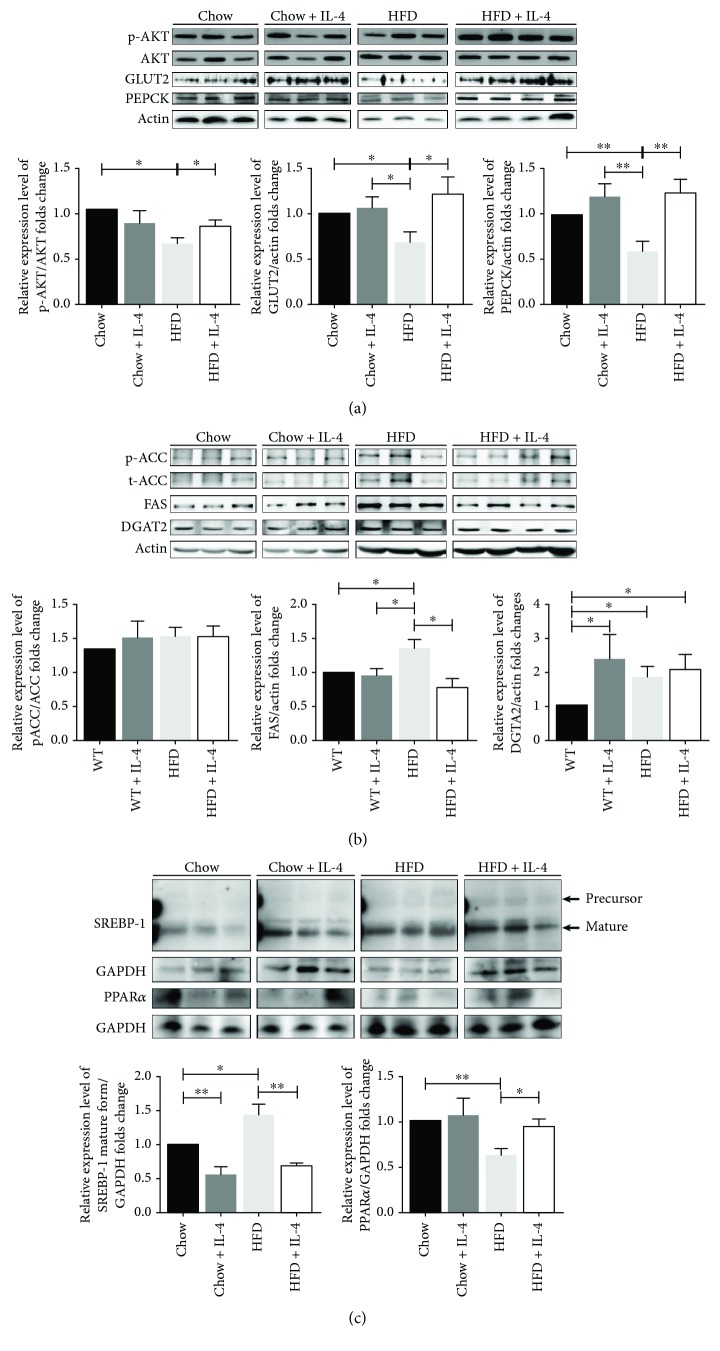
IL-4 modulates genes critical for hepatic gluconeogenesis, fatty acid synthesis, and lipogenesis in chow and HFD mice. Western blot analysis and quantification of (a) p-AKT, total AKT, GLUT2, and PEPCK; (b) p-ACC, total ACC, FAS, and DGAT2; (c) SREBP-1 and PPAR*α* expressions in chow, chow + IL-4, HFD, and HFD + IL-4 mice. Values were given as means ± SEM for *n* = 5–9; ^∗^ *p* < 0.05; ^∗∗^ *p* < 0.01.

The *in vivo* effects of IL-4 on hepatic energy metabolism were further analyzed at the molecular level. Phosphorylated AKT (p-AKT) was significantly increased in HFD + IL-4 mice; whereas, no significant alterations of p-AKT between chow and chow + IL-4 mice were observed (Figure 5(a)). GLUT2 expression in HFD mice was significantly decreased (Figure 5(a)). Notably, IL-4 rescued the GLUT2 expression in HFD mice to a comparable amount to chow mice (Figure 5(a)). The elevated p-AKT and GLUT2 in the IL-4 treated mice suggest that IL-4 promotes hepatic insulin sensitivity via upregulating the critical insulin signaling molecular AKT and the glucose sensor GLUT2.

Hepatic gluconeogenesis plays a key role in the maintenance of systemic glucose levels. We, therefore, investigated the possible regulation of IL-4 to PEPCK, the critical enzyme for hepatic gluconeogenesis *in vivo*. Our results showed that PEPCK was diminished in HFD mice, whereas this phenomenon was attenuated in HFD + IL-4 mice (Figure 5(a)).

Levels of several key enzymes for fatty acid synthesis and lipogenesis were also analyzed. While no significant difference of p-ACC and FAS between chow and chow + IL-4 mice (Figure 5(b)) was detected, DGAT2, an important enzyme of triglyceride synthesis from diacylglycerol and acyl-CoA in the liver, was significantly increased in chow + IL-4 mice (Figure 5(b)). Compared with chow mice, FAS and DGAT2 in HFD mice were significantly increased by about 1.35 and 1.43 folds, respectively; however, only FAS was attenuated by IL-4 treatment in HFD mice (Figure 5(b)). In addition, IL-4 significantly downregulated the mature active form of SREBP-1 both in chow and HFD mice while only upregulated PPAR*α* in HFD mice (Figure 5(c)). These data suggest that under physiological condition, the increased hepatic adiposity and serum triglycerides under IL-4 treatment may result from the enhanced hepatic lipogenesis in chow mice. Collectively, the above data reveal that the net effect of IL-4 on hepatic energy metabolism is to facilitate the anabolic effects of insulin by promoting AKT signaling and lipogenesis.

## 4. Discussion

The correlation between inflammation and metabolic conditions is first addressed by Hotamisligil et al. They reported that TNF-*α* in adipocytes of obese animals is markedly increased, and TNF-*α* neutralization leads to a decrease of insulin resistance [2]. Since then, accumulating evidence shows the significant correlation between circulatory TNF-*α* and body mass index [37, 38]. Moreover, obesity is proved to be a state of chronic inflammation as indicated by increased plasma concentrations of various inflammatory mediators [39–41].

T2DM is characterized by elevated triglycerides, dyslipidemia, and insulin resistance [42]. While the study regarding obesity and inflammation is booming, Crook et al. [43] and Pickup et al. [4] proposed that T2DM is also an inflammatory condition. Later on, emerging evidence confirms their hypothesis that T2DM is associated with chronic inflammation. IL-6 is one of the most studied cytokines, which acts as a glucoregulatory hormone through multiple mechanisms. In hepatocytes, IL-6 inhibits insulin signaling and GS activities [19, 44], as well as promotes glucose output, glycogen phosphorylase activity, lipolysis, and triglyceride production [16, 45, 46]. In adipocytes, IL-6 increases basal glucose transport in a time- and dose-related manner [18]. In human, chronic subcutaneous administration of IL-6 induces hyperglycemia [47]. These multiple effects of IL-6 on metabolism lead to hyperglycemia and insulin resistance. On the other hand, administration of TNF-*α* or IL-1 to rats impairs insulin action on peripheral glucose consumption and hepatic gluconeogenesis [17, 45, 48–50]. Accordingly, elevated proinflammatory cytokines are involved in glucose metabolism and result in the decreased insulin sensitivity. Nevertheless, data regarding the interaction between other cytokines and glucose metabolism are limited.

Except for the well-recognized role as the energy reservoir, adipose tissue is also characterized as an endocrine organ by secreting adipokines. These adipokines actively mediate energy homeostasis in response to external signals, as well as control feeding, thermogenesis, and neuroendocrine function [51]. Among the adipokines, adiponectin and leptin modulate body weight and food intake through regulating AMP-activated protein kinase (AMPK) activity via binding to AdipoR1/2 and Ob-R in the hypothalamus [52, 53]. Our previous finding that IL-4 administration regulates the appetite-controlling adiponectin and leptin [22] may explain the decreased food intake and body weight in HFD mice with IL-4 treatment (Figure 4). Moreover, our most recent data regarding IL-4 reduced AgRP and NP-Y while increased POMC expression (unpublished data) further support the functions of IL-4 in mediating appetite and energy metabolism. Accordingly, IL-4 actively modulates feeding behavior and energy homeostasis by both directly regulating hypothalamus-derived appetite-controlling mediators and indirectly modulating adipokines.

In addition to the immunological functions, IL-4 is implicated to abrogate IRS-2-associated PI3K and GSK-3 activity in macrophages of the T2DM model [54, 55]. Our previous findings identified the participation of IL-4 in regulating metabolism, including the association between IL-4 promoter polymorphisms and clinical lipid parameters [20], improvement of insulin sensitivity and glucose tolerance [22], and suppression of lipid deposits in adipocytes [24, 25]. In this context, the present study further explored the regulation of IL-4 to hepatic glucose and lipid metabolism, including glucose uptake, glycogen synthesis, gluconeogenesis, FFAs, and triglyceride synthesis, for elucidating the roles of this anti-inflammatory cytokine in energy metabolism and the interplay between immune responses and metabolism.

In hepatic glucose metabolism, IL-4 exhibits insulin-independent regulatory activity to potentiate basal glucose uptake by upregulating GLUT2 (Figure 2(a)). The core insulin signaling molecule AKT inactivates GSK-3*α*/*β* activity by phosphorylating GSK-3*α*/*β* at Ser9/21 to promote glycogen synthesis in hepatocytes. IL-4 alone does not cause apparent changes of p-AKT and p-GSK-3*β*; however, it exhibits synergistic effect on insulin signaling by boosting glucose uptake activity (Figure 2(b)). IL-4 also aids insulin-induced glycogen synthesis by attenuating GSK-3*α*/*β* activity (Figure 2(a)). Notably, regulation of IL-4 to GSK-3*α* is relatively more prominent than the *β* isoform although the alteration of p-GSK-*α*/*β* showed a parallel pattern upon IL-4 treatment. This observation is in support of the conclusion from Sung et al. that GSK-3*α* is the major regulatory molecule of glycogen synthesis in hepatocytes [56]. IL-4 treatment does not cause prominent changes on the rate-limiting gluconeogenic enzyme PEPCK. The above results implicate that IL-4 enhances glucose uptake via insulin signaling with no glucose output-mediating activity in hepatocytes. Accordingly, IL-4 aids insulin efficacy for promoting glucose uptake which, at least in part, accounts for the molecular mechanisms for our previous findings that IL-4 improves glucose tolerance and insulin sensitivity [22]. Moreover, these results reveal a similar scenario of IL-4 in mediating glucose metabolism in muscle cells (our unpublished manuscript) and lipid metabolism in adipocytes [24]. Instead of acting dominantly in regulating energy homeostasis, IL-4 facilitates insulin signaling and efficacy in energy metabolism.

Insulin resistance leads to hyperinsulinemia, increased hepatic glucose production, and decreased glucose metabolism. Our animal results showed that HFD mice exhibit higher serum insulin concentration (data not shown), whereas no differences were observed in the serum glucose level (Figure 4(d)). In addition, PEPCK is significantly reduced in HFD and induced in HFD + IL-4 mice (Figure 5(a)). We assume that HFD mice are in a prediabetic stage which evolves with HFD feeding duration and eventually leads to the diabetic onset. Another possibility is that the acute HFD exposure may lead to decreased PEPCK for providing the complementary metabolic protection during the early stage of excess caloric supply [57–59]. We speculate that the discrepancy between the *in vitro* and *in vivo* data may be originated by the reduced *de novo* gluconeogenic capacity in HFD mice in response to excess nutrition supply [60, 61], while IL-4 administration promotes PEPCK and thus the gluconeogenic activity via improving hepatic insulin sensitivity.

As for hepatic lipid metabolism, IL-4 upregulates genes promoting triglyceride synthesis, including FAS and DGAT2, and suppresses ACC1 phosphorylation (Figure 2(d)). In addition, mice with regular diet and IL-4 administration show higher circulatory triglyceride levels (Figure 4(d)). These observations reveal that IL-4 upregulates *de novo* lipogenesis in hepatocytes under physiological condition, which is further supported by the *in vitro* results that concomitant insulin and IL-4 exposure leads to increased FFA uptake and triglyceride contents (Figure 3). These findings are further verified by the animal experiments in which the hepatic adiposity is increased from mice with long-term IL-4 administration (Figures 4(e) and 4(f)). Our previous report reveals that circulatory adiponectin, leptin, and FFAs are elevated in AdIL-4 mice with transient IL-4 overexpression; meanwhile, FFA levels are also increased in HFD mice with long-term IL-4 injection [16]. Since adiponectin is known to improve insulin sensitivity and inhibit hepatic gluconeogenesis [62], we suggest that the metabolic effects of IL-4 may be exerted both by its direct signaling and indirect influences on other metabolism-mediating agents such as adiponectin. Taken the above results together, we conclude that the net effect of IL-4 is to improve insulin sensitivity by promoting hepatic energy deposits through upregulating glucose uptake and lipid synthesis.

Despite the positive role of IL-4 in metabolism, we previously showed that the prolipolytic activity of IL-4 leads to elevated blood FFAs [22, 24]. Intriguingly, while transient IL-4 does not cause prominent changes of hepatic adiposity (AdIL-4 mice, Figure 4(e)), hepatic lipid contents are significantly increased in mice receiving long-term IL-4 (HFD + IL-4 mice, Figure 4(f)). Therefore, although IL-4 exhibits positive regulatory effects to boost insulin efficacy, the energy deposit-promoting activity of IL-4 may make the liver more susceptible to develop steatosis under insulin resistant and diabetic models. This may explain our previous observation of the fatty liver in HFD + IL-4 mice [22]. We infer that under the status of consistent overnutrition, the excess circulatory FFAs released by peripheral tissues (such as adipose tissue due to the prolipolytic effect of IL-4) are transported to the liver [63] to result in the consequence of steatosis.

**Figure 6:**
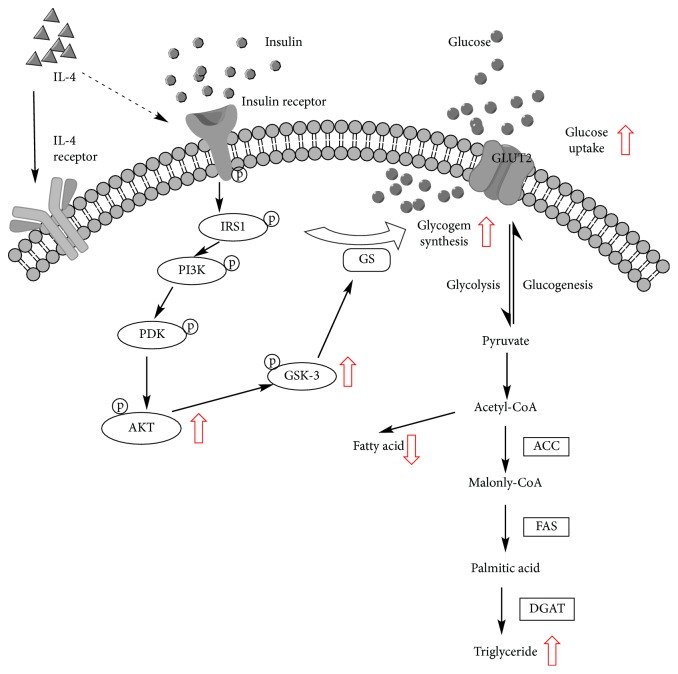
Regulation of hepatic glucose and lipid metabolism by IL-4. IL-4 stimulates AKT and GSK-3*α*/*β* phosphorylations, which in turn upregulates glucose uptake and GS activity for promoting hepatic glycogen synthesis. In addition, IL-4 promotes hepatic triglyceride contents through enhancing FFA uptake and the expression/activity of lipogenic enzymes, including ACC, FAS, and DGAT2. The net effects of IL-4 on hepatocytes are to promote energy storage by enhancing insulin-stimulated glucose uptake and lipid synthesis under physiological condition.

The present study elucidates the novel function of IL-4 in regulating hepatic glucose and lipid metabolisms. A model for the IL-4 modulating hepatic energy metabolism is illustrated (Figure 6). IL-4 stimulates Akt and GSK-3*α*/*β* phosphorylations, which in turn upregulates glucose uptake and GS activity for promoting glycogen synthesis. In addition, IL-4 promotes hepatic triglyceride contents through enhancing FFA uptake and the expression/activity of lipogenic enzymes, including ACC, FAS, and DGAT2. The major effects of IL-4 on hepatocytes are to promote energy storage by enhancing insulin-triggered glucose uptake and *de novo* lipid synthesis under physiological condition. Therefore, better glucose tolerance and insulin sensitivity are achieved by the capacity of IL-4 to synergize insulin signaling. Nevertheless, under insulin resistant status, the hepatic lipogenesis-promoting activity of IL-4 renders the liver more susceptible to develop steatosis by increasing FFA uptake and endogenous lipid synthesis. Therefore, imbalanced cytokine production might result in deterioration of metabolic chaos in patients suffering from diabetes with hyperglycemia.

Our study proves that the anti-inflammatory cytokine IL-4 improves insulin efficacy and modulates energy metabolism of insulin-targeting organs through multiple functions. IL-4 harbors antilipogenic ability by suppressing adipocyte differentiation and promoting lipolysis in mature adipocytes [24]. IL-4 potentiates basal glucose uptake and enhances insulin-induced glucose uptake in muscle cells (our unpublished manuscript). In the present study, we demonstrate that IL-4 boosts insulin-induced energy deposits in hepatocytes by upregulating glucose uptake and lipogenesis. Hopefully, the above findings not only provide new insights regarding the roles of IL-4 in metabolism and the interaction between cytokine and insulin but also add the clues to the underlying mechanism leading to metabolic abnormalities.

## Data Availability

Previously reported data were used to support this study and were cited at relevant places within the text as references [20–25].

## Abbreviations

2-NBDG: 2-[N-(7-nitrobenz-2-oxa-1,3-diazol-4-yl)amino]-2-deoxy-D-glucose
ACC1: Acetyl-CoA carboxylase 1
AdIL-4: Adenovirus containing full-length IL-4
ALT: Alanine transaminase
DGAT2: Diglyceride acyltransferase 2
FAS: Fatty acid synthase
GATA3: GATA-binding protein 3
GLUT2: Glucose transporter 2
GS: Glycogen synthase
GSK-3: Glycogen synthase kinase 3
GAPDH: Glyceraldehyde 3-phosphate dehydrogenase
FFAs: Free fatty acids
HDL-C: High-density lipoprotein-cholesterol
HFD: High-fat diet
IL: Interleukin
IL-4R*α*: Interleukin-4 receptor *α* chain
INS: Insulin
PEPCK: Phosphoenolpyruvate carboxykinase
PPAR*α*: Peroxisome proliferator-activated receptor alpha
SREBP-1: Sterol regulatory element-binding protein 1
SF: Serum free/serum starvation
STAT6: Signal transducer and activator of transcription 6
STZ: Streptozotocin
T2DM: Type 2 diabetes mellitus
TNF-*α*: Tumor necrosis factor-*α*.

## Supplementary Materials

Supplementary Materials Supplementary Figure 1: four-week-old male C57BL/6 mice were fed with high-fat diet (HFD) or standard chow diet and i.p. administered with recombinant IL-4 (1000 pg per mouse) every other day for 8 weeks [22]; then glucose tolerance test (GTT) and insulin tolerance test (ITT) were conducted. The data showed that IL-4 administration exhibited better glucose tolerance and insulin sensitivity in HFD mice.
