# Supplementary material for: Interleukin-4 Boosts Insulin-Induced Energy Deposits by Enhancing Glucose Uptake and Lipogenesis in Hepatocytes
Source: Oxid Med Cell Longev. 2018 Nov 21;2018:6923187. doi: 10.1155/2018/6923187 (PMC6280305; doi:10.1155/2018/6923187)
Supplement: Supplementary Materials — Supplementary Figure 1: four-week-old male C57BL/6 mice were fed with high-fat diet (HFD) or standard chow diet and i.p. administered with recombinant IL-4 (1000 pg per mouse) every other day for 8 weeks [22]; then glucose tolerance test (GTT) and insulin tolerance test (ITT) were conducted. The data showed that IL-4 administration exhibited better glucose tolerance and insulin sensitivity in HFD mice. [file 6923187.f1.pdf]

Supplementary Figure 1

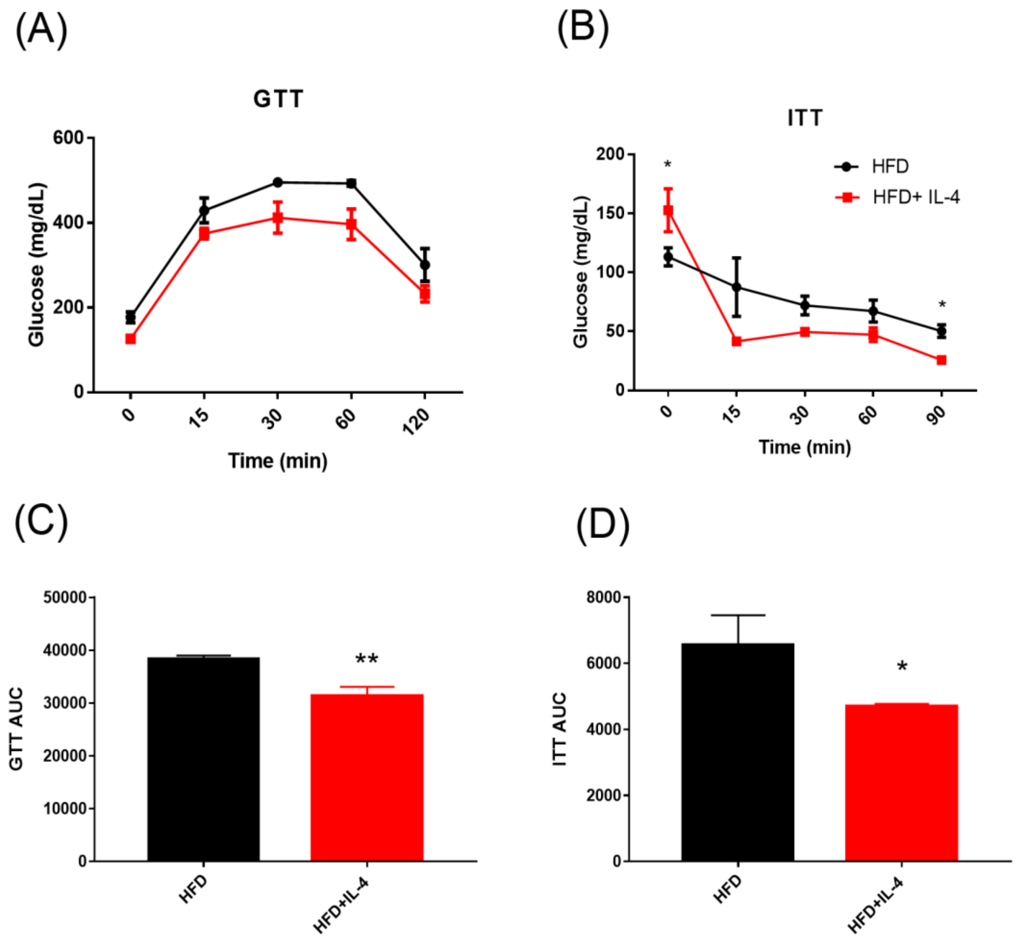

Supplementary Fig. 1 GTT and ITT in HFD and HFD+IL-4 mice.
(A): GTT (2g/kg glucose) in 10 hours fasted HFD and HFD+IL-4 mice. (B): ITT (0.75 units/kg insulin) in 10 hours fasted HFD and HFD+IL-4 mice. (C) and (D): Area under the curve (AUC) for HFD and HFD+IL-4 mice over course of GTT and ITT was calculated and is expressed as arbitrary units. Data are mean ± SEM. n = 5/group. *p<0.05 and **p<0.01 v.s HFD control.
